# AI-Based Grading of Facial Aging Signs and Prediction of Visible Age From Standardized Facial Images: Algorithm Development and Internal Evaluation Study

**DOI:** 10.2196/93236

**Published:** 2026-10-28

**Authors:** Frederic Flament, Panagiotis-Alexandros Bokaris, Tao Li, Aurelie Abric, Sileye Ba, Julien Despois, Hussein Jouni, Laudine Bertrand, Paul Michel, Thomas Dauxerre, Diala Haykal, Hughes Cartier

**Affiliations:** 1 L'Oréal Research and Innovation, 9 rue Pierre Dreyfus, Clichy, 92110, France, 33 149795334, 33 149795142; 2 Eurosyn, Villebon-sur-Yvette, France; 3 Centre Médical Laser Palaiseau, private practice, Palaiseau, France; 4 Centre Médical Saint Jean, Arras, France

**Keywords:** AI, visible age, biological markers, longevity, image analysis, clinical grading, facial signs

## Abstract

**Background:**

Facial appearance influences visible age (VA), defined here as the age perceived by others; this perception-based outcome differs from chronological age and biological age. Human evaluation of facial aging signs can be time-consuming and subject to interobserver variability. A standardized AI-based approach might offer a more reproducible assessment of visible facial aging in digital dermatology research.

**Objective:**

This study aimed to develop and evaluate an AI-based process for automatically grading facial aging signs and predicting the VA of participants from standardized full-face images.

**Methods:**

Evaluation of full-face photographs of 468 North American women aged 18 to 80 years recruited in Dallas and Phoenix, Texas. Fifteen trained experts, including dermatologists, graded 13 aging signs using Skin Aging Atlas scales. A separate, naïve group of 253 North Americans aged 20 to 60 years, recruited in New York City, estimated the VA of each participant. For each sign, facial areas were pulled out using facial landmarks and fed into ResNet-based regression models, which were trained to match the averaged expert scores. The automated facial sign grading models were tested on 90% of the image set, and the remaining 10% was used for evaluation. Results were assessed using the mean absolute error, the proportions of estimates within 0.5, 1.0, and 1.5 grading units of the expert reference, and Spearman correlations between chronological age, human-assessed VA, and predicted visible age (PVA).

**Results:**

The mean absolute error was below 0.3 grading units for 11 of the 13 facial aging signs. The average proportion of automatic gradings within 1 grading unit of the expert reference was about 98% for participants of East Asian ancestry group, 96% for participants of African ancestry group, and 97% for participants of European ancestry group. Human-assessed VA correlated strongly with real age (*r*=0.94, 95% CI 0.928‐0.950; *P*<.001), although participants were perceived as around 10 years older than their real age, on average. The AI-PVA also correlated strongly with the human-assessed VA (*r*=0.93, 95% CI 0.917‐0.941; *P*<.001), with approximately 70% of estimates within 5 years.

**Conclusions:**

The AI-based system showed low grading errors across most facial aging signs and also a fairly strong relationship between the PVA and human-assessed VA under standardized image acquisition conditions. Consequently, the system could provide a reproducible digital outcome for facial aging research and future longitudinal monitoring. However, external validation across separate populations, in men, and across image acquisition devices is required before clinical and consumer-facing implementation. As the predicted visible age is derived from quantified clinical facial signs, it reflects measurable features of visible facial aging, while its relationship with biological age and overall health remains to be established through future studies integrating biological biomarkers.

## Introduction

The lifespan of humans, otherwise called life expectancy, has shown a rather constant and significant progression in recent decades. According to the World Health Organization, global life expectancy increased from 66.8 years in 2000 to 73.1 years in 2019, and healthy life expectancy increased from 58.1 years to 63.5 years throughout the same time frame [1]. These figures vary, however, widely among adults with regard to genetic inheritance, exposure to life-threatening diseases, and geography. This large diversity introduces the notion of health span, that is, the years lived in good health, free from chronic disease and functional decline, including mental health [2-4].

This has reshaped the frontiers of the science of longevity, that is, the use of integrative strategies involving cosmetics, devices, digital services, coaching, oral supplements, and so on, far beyond a mere topical antiaging cosmetic solution. Facial skin, as a marker of the aging process, whether chronological and/or photoinduced, displays different aspects over decades and is an important element of health span, including well-being [5-8]. Previous studies have shown that these changes vary substantially across ethnic ancestries and genders [9-12]. Especially as a mirror of inner and invisible changes, facial skin could pave the way to exploring new holistic skin “clocks.” Yet, in the absence of biological measurements, visible facial aging should not be deemed equivalent to biological aging.

In this framework, different notions of age need to be differentiated. Real age (RA) refers to chronological age, whereas visible age (VA) refers to more or less the age perceived by others from facial appearance. Predicted visible age (PVA), as used in the current study, refers to an algorithm-derived estimate of the same VA. Meanwhile, biological age represents physiological or molecular aging and typically depends on biological markers. Although these dimensions seem related, they are not quite interchangeable. Previous work has already shown that perceived age can be linked with age-related phenotypes, functional status, molecular markers, and survival, supporting its interest as a perception-based aging outcome [13,14]. Recently, deep learning methods have likewise suggested the possibility of predicting perceived facial age from facial images [15].

Within this evolving paradigm, cosmetic dermatology is thus emerging as more than a discipline of aesthetic refinement, and it has become a meaningful participant in promoting health span and contributing to broader integrative longevity strategies. VA (eg, age as perceived by others) may, therefore, represent a digital health and perception-based outcome. The extent to which VA mirrors biological age and could be compared with RA (chronological age, as presented on an ID card) is one facet, among others, of understanding individual aging and relationships with patients and consumers for all actors [16-27]. The present study does not investigate VA as a measure of biological age. In short, fitting the VA of a participant with some biological markers of the skin is an ambitious project that could ideally be performed in real-life conditions, such as points of sale (POS) for cosmetic products or clinics with doctors and practitioners. From a technical viewpoint, recent years have shown fruitful help from smartphones as image collectors, thanks to their efficient cameras and high resolution (≥5 megapixels). Smartphones equipped with adequate algorithms are increasingly used and can allow the prompt analysis of images from very large cohorts of participants in real life or at home [28-31]. Such image-based approaches are of special interest in digital dermatology, where more standardized and reproducible evaluation of visible facial features could complement conventional clinical or expert grading.

As for the biological aspect, the recent introduction of systems for fast assessments of biomarkers will hopefully yield valuable data related to the root causes of skin aging, viewed as longevity “clocks.” Proteomic patterns, for example, can be extracted from adhesive patch sampling of the skin surface [32] and link invisible and visible aging manifestations. As these kinds of approaches are currently used to establish biological age, fitting their future results with a PVA remains instrumental in developing the most coherent ecosystem to accompany patients and consumers in engagement, compliance, and long-term monitoring. Hence, such integration represents a future perspective, as no biological markers were evaluated in the current study.

Although there is growing interest in AI-based facial assessment, approaches to combining standardized facial image capture, expert grading, and independent human assessment of VA are still needed. The question remains: do we really get more reliable and less variable results when using automatic grading? To increase diversity within the development set, the study included North American women spanning various ages and ethnic backgrounds, and covered a broad range of facial skin tones and visible aging indicators. Basically, the work aimed to design and test an AI-driven pipeline for automatically scoring facial aging signs and estimating VA using standardized full-face photographs. It also intended to compare this automatic grading with expert assessments and examine the relationships among RA, human-assessed VA, and AI-PVA across the ancestry groups represented in the study [33-35].

## Methods

### Study Design

The study followed a 5-fold structure: the acquisition of standardized full-face images from a cohort of North American women; the grading of visible facial signs by a panel of trained experts; the building of an AI-based automatic grading system; the evaluation of VA by an independent, naïve panel; and, finally, the calculation of the PVA.

### Ethical Considerations

The study was approved by L'Agence nationale de sécurité du médicament et des produits de santé (ANSM, France) under reference number 2025-A02830-49. Oral and written consent, in accordance with the Declaration of Helsinki, was obtained from all participants related to the study objectives and procedures. The consent also included that images would be taken under anonymized conditions, blind-coded, and deleted after study completion. Participants also consented to the use of their images for publication.

### Panel of Full-Face Photography

A total of 468 North American women (aged 18–80 y) were selected through a local agency and photographed in local facilities (Dallas, Texas, and Phoenix, Arizona, USA) using the Nexa device (Canfield Beauty) for standardization (position, lighting, etc). Figure 1 illustrates the device and 3 examples of the photographs provided, showing ethnic backgrounds and skin tones. Two inclusion criteria were used: (1) age between 18 and 80 years and (2) absence of any facial disorder (scars, acne, melasma, seborrheic dermatitis, telangiectasia, etc). The panel consisted of participants of different ages and ethnic ancestries, as presented in Table 1.

**Figure 1.**
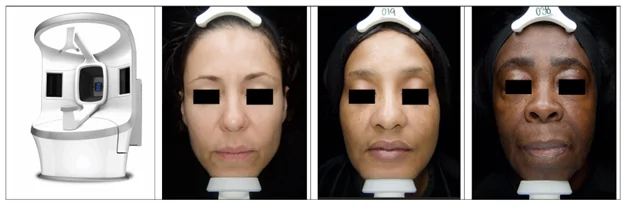
The Nexa device and 3 examples of full-face images from different skin tones.

**Table 1.** Distribution of the 468 North American women with clinical standardized pictures dataset according to ethnic ancestries and age groups.

Ethnic ancestry	18-30 y, n	31-40 y, n	41-50 y, n	51-60 y, n	61-70 y, n	71-80 y, n	Total, n
East Asian	16	16	14	26	20	8	100
African ancestry	28	25	23	15	28	6	125
European	52	56	48	29	42	16	243
Total	96	97	85	70	90	30	468

### Panel of Trained Experts for Grading Facial Signs

A panel of 15 trained experts (including dermatologists), previously used on different occasions for comparable tasks, was asked to grade the severity of 13 facial signs (Table 2) according to reference scoring ranges obtainable from ethnic-specific skin atlases [36,37] under standardized conditions. Each expert grader was seated about 1 m away from a 24-inch high-resolution display with a resolution of 1920 × 1200 pixels. The screen was calibrated daily using a colorimeter. To ensure robustness of this process, several images were presented twice during the evaluation process without being identified as duplicates. The high number of experts allowed for the exclusion of graders in cases of erratic scores.

**Table 2.** Thirteen clinical facial signs and their respective photographic scale’s ranges.

Facial signs	Definition	Scale range
Forehead wrinkles	Depth of the transverse wrinkles on the forehead	0‐8
Glabellar wrinkles	Depth of vertical wrinkles between eyebrows	0‐6
Crow’s feet wrinkles	Depth of wrinkles at the area of the outer eye corner	0‐6
Periorbital wrinkles (upper cheek area)	Depth of folds at malar area below Crow’s feet, eye orbit excepted	0‐9
Nasolabial fold	Depth of the fold present between the base of the nose and lips	0‐7
Marionette lines	Depth of folds at the corner of lips	0‐8
Pigmentary spots (density)	Number of spots per area unit on the cheek	0‐7
Ptosis of lower part of the face	Sagging severity of the lower parts on each side of the chin	0‐6
Diffuse redness	Diffuse redness and microvessels visible, especially on cheeks	0‐4
Cheek skin pores	Size of visible pores on the cheek irrespective of their densities	0‐5
Interocular wrinkles	Depth of horizontal folds between inner eye corners	0‐7
Perioral lip wrinkles	Depth of wrinkles on superior labial area	0‐6
Eye bags	Intensity of the ocular bulge	0‐6

For each facial sign and photograph, the mean of the scores given by the experts was used as the reference score. Despite using integer grades on the photographic scales, the averaged expert scores provided continuous reference values and enabled the automatic system to account for subgrade variations.

### Naïve Panel Attributing VA to Photographs

A total of 253 North American women of different ethnic backgrounds were recruited in New York City, USA, as a naïve panel by a local agency dedicated to part-time occupations. These participants were aged 20 to 60 years, with 48% (n=121) aged between 20 and 39 years and 52% (n=132) aged between 40 and 60 years, with good visual acuity as a unique inclusion criterion. They were informed about the objective of their task and signed an informed consent form, ensuring full anonymity. The whole panel, when successively viewing the 468 blind-coded photographs presented at random, was asked to answer a simple question: “How old is this woman?” Their answers then referred to VA and were linked to their RA.

### AI-Based Automatic Grading System

Based on the expert’s grading, an AI-based automatic grading system was developed to automatically compute the severity of the 13 facial signs. To assess facial clinical signs with high accuracy, the AI-based automatic grading system is framed as a regression task, where a dedicated model is trained to estimate a continuous severity score for each targeted clinical sign from a cropped facial image.

First, an off-the-shelf landmark detection algorithm is run to compute key points used as reference landmarks on the face, such as the tip of the nose, inner corners of the eyes, etc. Based on these landmarks, a cropping step uses handcrafted heuristics to extract the relevant zones of the face as defined by the clinical grading atlases.

The chosen backbone was ResNet, a streamlined convolutional neural network using residual connections to improve prediction accuracy [38,39]. The architecture consists of 5 convolutional residual blocks, progressively reducing the spatial dimensions to a 1 × 1 × 512 feature map, followed by 3 fully connected layers with rectified linear unit (ReLU) activations that reduce the output dimensionality to a single scalar (the predicted clinical severity score). Each of the 13 facial signs was handled by a dedicated model instance, resulting in a holistic multistep diagnostic pipeline that takes a full-face image as input and yields a list of sign-specific predicted scores as output.

Functionally, each model takes as input a crop of the image that corresponds to the target clinical sign (eg, forehead wrinkles, periorbital wrinkles, and marionette lines) and predicts a scalar value within skin aging atlas grading scales (Table 2) [36,37]. The average grades provided by 15 experts were used as reference labels. Although the scales are defined with integer values (grade 0, grade 1, etc), the averaged expert annotations allowed the models to account for subgrade variations. The pooled labeled data from 15 expert annotators, trained to evaluate with high precision, allow us to account for subgrade accuracy by targeting the average prediction.

### Model Training and Internal Evaluation

Each model was trained using the following configuration:

- Loss function: difference between the predicted score and the average of expert annotations
- Optimizer: Adam [40] with β_1_=0, β_2_=0.99
- Learning rate: 0.0005
- Batch size: 32
- Training duration: 300 epochs

To prevent overfitting and maximize generalizability, two complementary strategies were used. First, early stopping was monitored using an internal validation subset, carved out from the training data (10% of the 90% training subset), with training halted if validation loss did not improve over a defined patience window. Second, data augmentation was systematically applied during training, including the addition of chromatic noise, shifts in hue and lighting intensity, and small random rotations of input crops. These augmentations simulated real-world variability in capture conditions (eg, minor lighting inconsistencies and slight head movements) and reduced the risk of overfitting to the controlled photographic conditions of the training set, thereby improving robustness when deployed in live settings.

An automatic grading system was trained and evaluated on a standardized dataset that had been scored by experts and dermatologists. The dataset was split into training and test subsets, with 90% of the data used for training the model and 10% used to evaluate the capacity of the system to generalize to images not encountered during training [22,33]. Within the 90% training subset, a further internal validation split (10% of the training data) was used exclusively for early stopping and model selection purposes, while the remaining training data were used for model fitting. The split was stratified by ethnic ancestry and age group to ensure balanced representation across subgroups. Participant-level separation was strictly enforced to prevent any form of data leakage.

To ensure the resulting system is as robust and accurate as possible, the training procedure includes a data augmentation step, where input crops are randomly degraded or altered to teach the system to ignore such perturbations. Despite the use of controlled lighting and poses, data augmentation minimizes the risk that unforeseen circumstances degrade the performance of the system. The data augmentation includes the addition of chromatic noise, shifts in hue and lighting, as well as small rotations of the crops.

The crops are fed into the corresponding models to obtain a predicted grade of clinical severity, thereby linking with PVA. This results in a holistic algorithm that functions as a multistep diagnosis tool, taking a full-face image as input and yielding a list of predicted scores as the output.

### PVA Derivation

Based on the validated automatic grading outputs, PVA was computed from the 10 facial signs found to be significantly correlated with VA as assessed by the naïve panel (Table 3; cheek skin pores (CSP), pigmentary spots (PS) density, and diffuse redness (DR) were excluded due to low correlation with VA). For each retained sign *i*, a second-order polynomial regression of the form *Y* = *A*_*i*_*x*_*i*_^2^ + *B_i_x_i_* + *C_i_* was fitted using ordinary least squares on the training subset exclusively, where *x_i_* was the automatically predicted facial sign grade, *Y* was the associated VA estimate, as assessed by the naïve panel, and *A_i_*, *B_i_*, and *C_i_* were the regression coefficients. The PVA of each photographed participant was obtained by averaging the polynomial outputs across all 10 retained signs. Coefficients *A_i_*, *B_i_*, and *C_i_* for each sign, as derived from the training set, are reported in Table 4. The generalization performance of the PVA model was then assessed on the held-out test subset by computing the Spearman correlation between the PVA and the VA assessed by the naïve panel (N=468), yielding *r*=0.93 (*P*<.001). This strict train/test separation ensures that the reported correlation reflects a genuine out-of-sample prediction and provides a robust safeguard against overfitting.

**Table 3.** Overall agreement statistics for the extended facial sign/imaging condition analyses.

Facial sign/imaging condition	MAE^a^	RMSE^b^	ICC^c^ (95% CI)	Percentage within 1 grade (%)
Crow’s feet wrinkles (n=173)	0.314	0.478	0.949 (0.930-0.960)	93
Eyebags (n=173)	0.184	0.365	0.906 (0.870-0.930)	96
Pigmentary spots (polarized; n=186)	0.879	1.080	0.659 (0.130-0.840)	66
Pigmentary spots (nonpolarized; n=186)	0.226	0.306	0.954 (0.940-0.970)	98
Cheek skin pores (n=186)	0.239	0.339	0.895 (0.860-0.920)	98
Marionette lines (n=186)	0.218	0.321	0.976 (0.970-0.980)	98
Forehead wrinkles (n=92)	0.278	0.359	0.937 (0.910-0.960)	98
Glabellar wrinkles (n=92)	0.240	0.468	0.912 (0.870-0.940)	98
Interocular wrinkles (n=92)	0.401	0.544	0.909 (0.860-0.940)	96
Nasolabial fold (n=186)	0.215	0.338	0.970 (0.960-0.980)	96
Ptosis (n=92)	0.303	0.466	0.886 (0.830-0.920)	93
Periorbital wrinkles (n=186)	0.201	0.283	0.967 (0.960-0.970)	99
Perioral lip wrinkles (n=92)	0.341	0.493	0.878 (0.820-0.920)	93
Diffuse redness (nonpolarized; n=92)	0.204	0.258	0.948 (0.920-0.970)	100
Diffuse redness (polarized; n=92)	0.177	0.218	0.963 (0.940-0.980)	100

a MAE: mean absolute error.

b RMSE: root mean squared error.

c ICC: intraclass correlation coefficient.

**Table 4.** Definition of coefficients for the equation for predicted visible age (PVA) based on facial clinical signs.

Facial signs/coefficients	A	B	C
Crow’s feet wrinkles	−1.43	18.07	14.13
Eye bags	−4.70	24.26	34.38
Marionette lines	−1.07	12.13	34.22
Forehead wrinkles	−1.78	15.59	33.33
Glabellar wrinkles	−2.75	20.06	32.19
Interocular wrinkles	−1.60	16.03	28.73
Nasolabial fold	−1.55	14.90	34.99
Ptosis of the lower part of the face	−2.22	18.45	31.89
Periorbital wrinkles	−2.10	19.04	27.57
Perioral lip wrinkles	−3.27	22.82	30.71

### Statistics

The level of agreement between the grades provided by the AI-based system and the experts was analyzed globally and stratified by ethnic ancestry based on metrics including MAE, root mean squared error (RMSE), intraclass correlation coefficient (ICC), Bland-Altman analysis, and calibration statistics. The number of paired observations varied by sign, reflecting the imaging protocol (not all signs were captured for every participant at every session). MAE was defined as the mean of the absolute differences between the automatically predicted grades and the averaged expert reference grades. For each facial sign, the proportions of individual predictions with an absolute error below 0.5, 1.0, and 1.5 grading units were also generated.

The correlations between PVA and VA, on the one hand, and PVA and RA, on the other hand (2 paired methods to be compared), were computed. Their significance was assessed using Spearman coefficient correlation tests overall, considering all photographed volunteers (N=468).

## Results

### Assessment Panel

The standardized photographic dataset consisted of 468 North Americans aged between 18 and 80 years old, including 100 women of East Asian ancestry, 125 of African ancestry, and 243 of European ancestry, spread across the different age groups, as shown in Table 1.

The expert grading panel comprised 15 trained experts, including dermatologists with previous experience in facial sign grading. The naïve panel included 253 North American women from different ethnic profiles, aged between 20 and 60 years old, with approximately 50% aged between 20 and 39 years and 50% aged between 40 and 60 years.

### Experts Panel Consensus and Variability

Table 5 illustrates the scoring dataset established by the expert panel, including MAEs calculated at different grade thresholds according to the Skin Aging Atlas classification. Most facial skin signs appear to be correctly assessed, with an acceptable MAE of around 0.5, except for periorbital wrinkles and the density of pigmentary spots (approximately 0.6).

**Table 5.** Values of the MAE in the scores of 13 facial signs recorded by 15 experts and dermatologists at different selected thresholds.

Facial sign	MAE^a^	Percent |error|<0.5	Percent |error|<1.0	Percent |error|<1.5
Panel A: East Asian ancestry				
Forehead wrinkles	0.44	66	93	99
Glabellar wrinkles	0.37	74	96	99
Crow’s feet wrinkles	0.52	58	88	97
Periorbital wrinkles	0.59	54	82	94
Nasolabial fold	0.44	67	90	97
Marionette lines	0.52	59	88	96
Pigmentary spots (density)	0.62	51	80	93
Ptosis of the lower part of the face	0.49	57	91	99
Diffuse redness	0.37	72	97	100
Cheek skin pores	0.52	57	87	97
Interocular wrinkles	0.55	55	86	96
Perioral lip wrinkles	0.43	68	92	99
Eye bags	0.45	66	90	98
Panel B: African ancestry				
Forehead wrinkles	0.46	63	90	98
Glabellar wrinkles	0.39	73	95	99
Crow’s feet wrinkles	0.53	57	87	96
Periorbital wrinkles	0.59	52	84	95
Nasolabial fold	0.49	65	88	96
Marionette lines	0.51	62	88	96
Pigmentary spots (density)	0.66	46	79	92
Ptosis of the lower part of the face	0.49	63	91	98
Diffuse redness	0.37	79	94	97
Cheek skin pores	0.59	53	82	94
Interocular wrinkles	0.51	57	88	97
Perioral lip wrinkles	0.47	62	91	99
Eye bags	0.43	69	92	97
Panel C: European ancestry				
Forehead wrinkles	0.48	63	89	97
Glabellar wrinkles	0.38	73	95	99
Crow’s feet wrinkles	0.49	61	89	98
Periorbital wrinkles	0.61	53	82	93
Nasolabial fold	0.45	65	90	97
Marionette lines	0.53	57	87	96
Pigmentary spots (density)	0.55	55	85	95
Ptosis of the lower part of the face	0.55	54	87	97
Diffuse redness	0.41	67	94	99
Cheek skin pores	0.49	60	90	98
Interocular wrinkles	0.53	57	88	97
Perioral lip wrinkles	0.45	64	92	98
Eye bags	0.39	73	93	98

a MAE: mean absolute error.

### Prediction Potential of the Automatic Scoring System

Table 6 summarizes the performance of the AI-based automatic grading system. On average across all 13 signs, considering the condition of MAE<1, which is the accepted error during a live evaluation by a clinician based on a standardized photographic scale, reliable accuracy is achieved for East Asian (~98%), African ancestry (~96%), and European (~97%) vs experts’ clinical ground truth.

**Table 6.** Performance of the AI-based automatic grading system.

Facial sign	MAE^a^	Percent |error|<0.5	Percent |error|<1.0	Percent |error|<1.5
Panel A: East Asian ancestry				
Forehead wrinkles	0.24	88	100	100
Glabellar wrinkles	0.18	94	100	100
Crow’s feet wrinkles	0.28	78	97	97
Periorbital wrinkles	0.21	90	100	100
Nasolabial fold	0.22	85	97	100
Marionette lines	0.23	87	95	100
Pigmentary spots (density)	0.25	87	95	100
Ptosis of the lower part of the face	0.27	88	94	100
Diffuse redness	0.15	100	100	100
Cheek skin pores	0.16	100	100	100
Interocular wrinkles	0.38	62	100	100
Perioral lip wrinkles	0.33	81	100	100
Eye bags	0.21	86	97	100
Panel B: African ancestry				
Forehead wrinkles	0.3	88	92	100
Glabellar wrinkles	0.4	77	92	96
Crow’s feet wrinkles	0.21	94	98	100
Periorbital wrinkles	0.17	94	100	100
Nasolabial fold	0.22	88	94	100
Marionette lines	0.18	94	98	100
Pigmentary spots (density)	0.24	84	96	100
Ptosis of the lower part of the face	0.29	81	92	96
Diffuse redness	0.2	100	100	100
Cheek skin pores	0.29	90	96	96
Interocular wrinkles	0.44	77	92	96
Perioral lip wrinkles	0.24	85	96	100
Eye bags	0.16	93	96	100
Panel C: European ancestry				
Forehead wrinkles	0.28	87	100	100
Glabellar wrinkles	0.17	96	100	100
Crow’s feet wrinkles	0.35	78	90	98
Periorbital wrinkles	0.22	86	99	100
Nasolabial fold	0.21	89	97	99
Marionette lines	0.24	91	99	100
Pigmentary spots (density)	0.21	92	100	100
Ptosis of the lower part of the face	0.31	81	96	100
Diffuse redness	0.18	98	100	100
Cheek skin pores	0.25	92	99	100
Interocular wrinkles	0.38	77	96	100
Perioral lip wrinkles	0.39	72	89	96
Eye bags	0.17	91	98	98

a MAE: mean absolute error.

Considering the average of all ethnicities, the MAE values were below 0.3 for 11 signs, except for interocular and perioral lip wrinkles, which showed higher values. Interestingly, the comparison of results between Tables 5 and 6 clearly indicates that the automatic grading system induces less variability and better reliability than that of the panel of trained experts, regardless of ethnicity or facial clinical sign. Indeed, the MAE values obtained suggest 2 or 3 times lower errors and variability for the automatic grading system compared with the experts’ panel.

### Additional Agreement Analyses

Additional analyses were conducted to further investigate how well the automatic grading system aligned with the expert assessments. Since pigmentary spots and vascular disorders were evaluated under both polarized and nonpolarized imaging conditions, these analyses included 15 facial aging sign/imaging condition pairings derived from the 13 facial signs assessed in the study.

Among these 15 sign/imaging condition combinations, the MAE was 0.292 grading units, and the RMSE was 0.419 grading units. The higher RMSE compared to MAE suggests the presence of some larger outlier prediction errors even though the average error remained low. Furthermore, more than 90% of the automatic predictions were within 1 grading unit of the expert reference for 14 of the 15 sign/imaging condition combinations. The main outlier was CPS under polarized conditions, where only 66% of the predictions were within 1 grading unit of the expert score.

The ICC values appeared to show good to excellent agreement for most of the evaluated signs. For CPS, especially under polarized lighting, the agreement was comparatively lower, with an ICC of approximately 0.66. Signs like upper lip wrinkles, ptosis, and cheek pores exhibited agreement within the good range, while most of the remaining sign/imaging condition combinations showed excellent agreement. The full ancestry-stratified statistics are provided in Multimedia Appendix 1.

Bland-Altman analyses showed mean biases close to zero for most signs, indicating little systematic overestimation or underestimation at the group level. Some proportional bias was observed at higher severity grades, whereas the limits of agreement were generally within approximately ±1 grading unit (Multimedia Appendix 2). Calibration plots indicate good linearity between the AI-automated grading and expert scores for most signs, with regression slopes close to 1.0 and intercepts close to 0. Similar to observations with higher MAE and lower ICC, deviations from the identity line were noted for CPS (polarized), whereas nonpolarized images demonstrated better-calibrated predictions for pigmentation-related signs (Multimedia Appendix 3).

### PVA

Figure 2 presents correlation coefficients between the 13 facial clinical grades predicted by the automatic grading system and the VA assigned by the naive panel. It clearly shows that most facial signs are interrelated in the perception of age, with the exception of CSP, PS (density), and diffuse redness (DR). They will, therefore, not be retained in the subsequent calculation of PVA.

Figure 3 presents correlation coefficients between RA and the ages perceived by a panel of 253 US women. Although these appear strongly correlated (*r*=0.94; *P*<.001), the 468 photographed women were perceived to be older than they are by about 10 years in a large majority.

**Figure 2.**
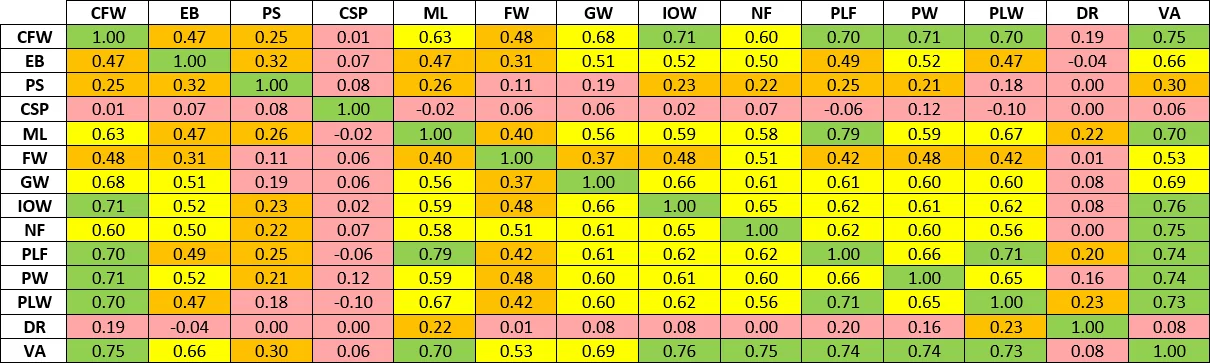
Correlation coefficients between all facial signs predicted by the AI-based automatic grading system and visible age (VA). The colored boxes show the levels of correlation with VA: green: ≥0.70; yellow: ≤0.5 and >0.7; orange: ≤0.2 and >0.5; red: ≥−0.1 and <0.2. CFW: crow’s feet wrinkles; CSP: cheek skin pores; DR: diffuse redness; EB: eye bags; FW: forehead wrinkles; GW: glabellar wrinkles; IOW: interocular wrinkles; ML: marionette lines; NF: nasolabial fold; PLF: ptosis lower part face; PLW: perioral lip wrinkles; PS: pigmentary spots; PW: periorbital wrinkles.

**Figure 3.**
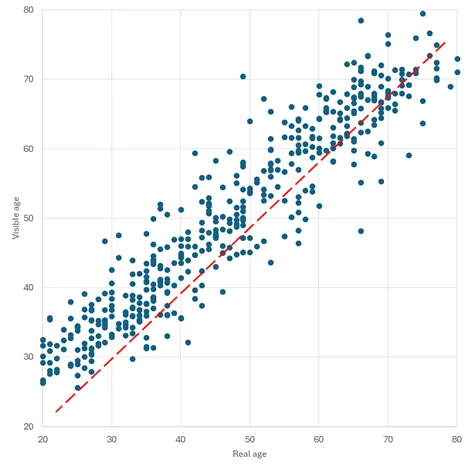
Correlation between the visible ages (assessed by the naïve panel) of the 468 US participants and their real ages (*r*=0.94).

For each of the 10 retained signs, a second-order regression of the type *Y* = *Ax*^2^ + *Bx* + *C* (where *x* is the grade established by the automatic grading system) is adopted, where *A*, *B*, and *C* coefficients of each of the retained signs are indicated in Table 4. The sum of all values of all *A*_*i*_*x*_*i*_^2 ^+ *B*_*i*_*x*_*i*_ + *C*, when averaged, allows obtaining the PVA of each of the 468 US participants.

PVA was strongly correlated (*r*=0.93; *P*<.001) with the VA evaluated by the naïve panel (Figure 4). Nevertheless, this figure shows that despite the good correlation, gaps were found within the 40- and 60-year age ranges. However, about 70% of assessments were within 5 years of the VA assessed by the naïve panel.

**Figure 4.**
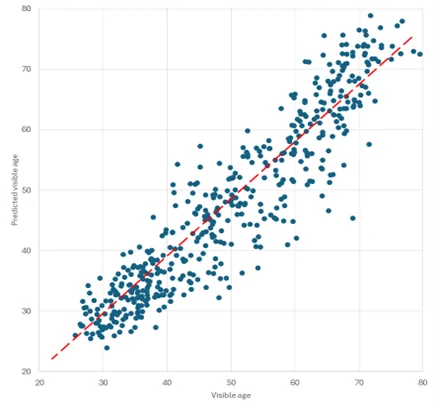
Correlation between predicted visible ages of the 468 US participants and their visible ages, as assessed by the naïve panel (*r*=0.93).

## Discussion

### Principal Findings

The present work aimed at analyzing how an AI-based process could predict the VA of a given participant from a standardized full-face photograph in a robust manner, while reducing the maximum of biases arising from human assessment. The study paired standardized image capture, clinical grading by experts, an independent naïve panel for VA assessment, and automatic grading of facial aging signs. The NEXA photographic system used in this study provided standardized image acquisition and good image quality together with its ease of use (rapidity, reproducibility, etc), that is, the primary conditions to allow the most precise analysis by a naïve panel, an expert group, or an automatic AI-based process. It is nevertheless clear that attributing a precise VA to a person from a single facial aspect is by no means an easy task. In real life, other criteria are used, such as hair presence or style, body positioning (straight or bent), walking pace, etc, as well as reflecting cultural, geographical, or environmental specificities. Facial traits remain, however, a suitable model of skin aging, abundantly studied as they integrate intrinsic biological changes and external impacts (sun exposure and lifestyles), as extensively reviewed [41].

A relatively low prediction errors for most facial signs were obtained when the results provided by the automatic system were compared with those from the expert panel. These findings were further confirmed by additional analyses, with good to excellent agreement obtained for most evaluated sign/imaging condition combinations. In the Bland-Altman analyses, the mean biases approached zero for most signs, and calibration overall was satisfactory. Still, performance was not homogeneous across all facial signs. Pigmentary spots exhibited lower agreement when assessed under polarized lighting. These findings suggest that the performance of the automatic grading system partially depends on the facial sign and imaging condition evaluated.

### VA and Human Perception

With regard to the assessments by the naïve panel, comparing VA and RA, although highly correlated (*r*=0.94; *P*<.001), leads to blurred results. Why this panel “viewed” almost all participants as much older (by 10 y or more at younger ages) than they really were is an open question [9,12]. In contrast to the blind/neutral feature of the automatic system, the perception of the panel is processed at a glance that rapidly integrates all facial aspects, which might attribute more “weight” to some facial regions or signs (upper or lower face, ptosis, etc) than others; this is a possibility that could be checked through an eye-tracking methodology (not explored here). To what extent such focus on specific facial regions obeys cultural or socioeconomic influences is another open question. In contrast, the automatic system provides, in the end, a realistic predictive estimate of the VAs of the 468 women studied, although Figure 3 shows larger gaps in the 40 and 60 years range, noting that these follow a normal Gaussian distribution. It is likely that such a result first illustrates the period when the process of facial aging leads to a larger variability among women with regard to hormonal changes (perimenopausal and postmenopausal periods). The latter indeed induce profound and widely varying effects among the women concerned in both physiological (skin included) and psychological status [42-45].

Second, the spontaneous attribution of a VA to a person likely differs in complexity according to age; that is, it is probably more precisely established at age ranges where markers of aging are either not very pronounced (18–30 y) or already settled (>60 y). It is noteworthy that 3 signs related to homogeneity (PS, CSP, and DR) showed low correlations with VA and were not included in the PVA calculation. When different ancestries are considered together, facial signs such as wrinkles and sagging may contribute more consistently to the overall perception of VA.

### Performance Across Ancestry Groups

The choice of performing the study in the United States enabled the inclusion of women from different ancestry groups and age ranges. The automatic grading system showed generally high agreement with expert assessments across the ancestry groups represented in the study. The additional analyses, however, showed variation depending on both facial sign and ancestry group. Hence, the overall performance estimates should not be read as exactly the same for every subgroup. In a diverse US female population, similar work had also demonstrated the feasibility of automated grading of facial signs using selfies, which depended on facial features, especially those associated with pigmentation [46].

These findings are important in the context of potential algorithmic bias. Although the inclusion of women of East Asian ancestry, African ancestry, and European ancestry increased diversity within the development dataset, it does not establish fairness or generalizability to all populations. In particular, performance across age groups was not separately characterized to the same extent as ancestry-related performance. Further evaluation on larger and more independent datasets will, therefore, be needed to assess whether performance stays comparable across the different demographic groups.

### Potential Relevance to Digital Dermatology and Health Care Setting

These results demonstrate that the application of standardized image analysis provides a reproducible digital outcome to assess visible facial aging. This approach might be useful for complementing traditional clinical grading by providing standardized quantitative assessment of facial aging signs and VA in the fields of dermatology and cosmetic dermatology.

Further potential applications could be found in the longitudinal monitoring of visible facial changes or in standardized assessments of appearance-related outcome measures in research and clinical practice. From this perspective, emerging concepts such as intelligent mirrors can offer a framework for the repeated, standardized, and noninvasive acquisition of skin phenotypes over time, supporting a transition from single time point assessments toward longitudinal skin monitoring [47]. These tools might additionally aid practitioners’, patients’, and consumers’ communication about visible change.

The present study has not evaluated diagnostic accuracy for diseases, nor has it explored the influence on clinical decisions, treatment outcomes, patient management, or health system outcomes. Thus, at this stage, the system cannot be regarded as being a diagnostic or prognostic tool. Furthermore, since the evaluation used standardized photographs captured with one imaging device, applicability for use at POS, in clinics, on smartphones, or in consumer-facing situations under less standardized conditions requires further demonstration.

The present approach may potentially be integrated with other measurement modalities (eg, cutaneous biomarkers) to explore relationships between visible facial aging and biological processes. However, such work represents a future research perspective.

### Ethical Considerations

While the application of AI-based facial grading and PVA in dermatology offers great promise, several ethical considerations must be addressed. First, more longitudinal studies are needed to validate the long-term imbrication between biological age and VA. While some treatments have been shown to influence biomarkers in the short term, their long-term impact on skin health and longevity remains unclear. Additionally, the use of biological age or VA as metrics for treatment decisions raises ethical questions, such as whether patients should be informed of their biological age if it differs significantly from their chronological age, and if so, how this information should be communicated alongside VA and how it might influence a patient’s perception. Another challenge lies in the accessibility of these advanced, expensive, and specialized tools in POS or clinics, limiting their availability to a broader patient population.

Addressing these barriers, for example, with nomad solutions on smartphones, is essential to ensure equitable access to these innovations, preventing disparities between patients who can afford high-end aesthetic or dermo-cosmetic treatments and those who cannot. Additionally, differences in life expectancy between men and women, influenced by hormonal and environmental factors, add complexity to longevity research. These variations underscore the importance of developing sex-specific approaches for personalized and effective treatments [48].

### Limitations and Future Perspectives

Several limitations of the study should be noted. The data are restricted to women in North America who were photographed with a standardized imaging device. Although women across various ages and ethnicities were included, this diversity does not, by itself, establish generalizability to men, populations outside North America, other image-capture devices, or less-standardized real-life conditions. Additionally, there was no validation of results on an independently collected dataset.

Further investigations will be necessary, including the use of independent datasets containing more varied human populations, as well as alternative image acquisition devices under variable imaging conditions, before broader clinical or consumer-facing applications can be considered.

Overall performance was satisfactory across all ancestry groups included in the study, with variations depending on the facial signs and subgroup. To further assess the robustness of, and possible biases associated with, each group, additional investigations would be necessary, as the current analyses are based on limited datasets that lack balance. The naïve panel consisted solely of North American women aged 20 and 60 years, and thus the VAs might partially represent their specificities and perceptions.

It is worth noting that the algorithm trained to predict VA is based on subjective human evaluations. As such, PVA reflects an assessment of visible changes related to chronological age rather than objectively measurable signs of biological aging. No biological, proteomic, or physiological markers were assessed in the present study, which further implies that while the idea of VA being a potential “skin clock” is appealing, its relationship with biological age, health span, or overall health should currently be considered exploratory. This AI-based process could, in future studies, be associated concomitantly with biological measurements extracted from the skin surface. This new tool will support engagement, compliance, and long-term monitoring. It is nevertheless clear that this system calls for some extensions, such as its application to men—for whom technical challenges regarding beards, for example, could be addressed in a new AI-based model—or a deeper study of countries’ specificities to tackle cultural shifts through larger cohorts of the major ethnic backgrounds.

In addition, building a global ecosystem to accompany patients and consumers through integrative longevity strategies is at hand, as we compare the MAE obtained here for the automatic grading system across all ethnicities (~97%) with that obtained with a similar system developed with Skin Aging Atlas references for smartphone selfies (~98%) [38,49]. This aspect will allow us to go further into North American women and controlled imaging. Another instrumental introduction will be leveraging technologies such as GANs for photorealistic and accurate personalized simulations [17,22,50] of future visible manifestations of longevity treatments of aging root causes—or the absence of them.

### Conclusions

The present study demonstrated that an AI-based process can automate the evaluation of some features of visible facial aging with relatively low prediction errors and provide a predicted VA that has a strong correlation with human-assessed VAs under standardized image acquisition conditions. The automatic grading system demonstrated good agreement with expert assessments across diverse ancestral backgrounds represented in the study, although performance varied by facial sign and subgroup.

These findings support the potential use of AI-based facial assessment as a reproducible digital outcome for facial aging research and longitudinal monitoring. Nevertheless, external validation in independent, more diverse, and less standardized conditions is required before any wider clinical or consumer-facing rollout. As predicted, VA is derived from quantified facial clinical signs; it reflects measurable features of visible facial aging, while its relationship with biological age, overall health, or its potential as a validated “skin clock” remains to be established through future studies integrating biological biomarkers.

## Supplementary material

10.2196/93236 Multimedia Appendix 1 Full agreement statistics stratified by ancestry group.

10.2196/93236 Multimedia Appendix 2 Bland-Altman plots.

10.2196/93236 Multimedia Appendix 3 Calibration plots.

## Abbreviations

CSP: cheek skin pores
DR: diffuse redness
ICC: intraclass correlation coefficient
MAE: mean absolute error
POS: points of sale
PVA: predicted visible age
RA: real age
ReLU: rectified linear unit
RMSE: root mean squared error
VA: visible age
