# Supplementary material for: AI-Based Grading of Facial Aging Signs and Prediction of Visible Age From Standardized Facial Images: Algorithm Development and Internal Evaluation Study
Source: JMIR Dermatol. 2026 Oct 28;9:e93236. doi: 10.2196/93236 (PMC13716349; doi:10.2196/93236)
Supplement: Supplementary file 1 [file derma-v9-e93236-s001.docx]

**Supplementary material I. Full Agreement Statistics Stratified by Ancestry Group**

Agreement statistics are shown for each facial sign/imaging-condition combination. ICC values are based on a two-way random-effects, single-measure, absolute-agreement model. Group labels have been harmonized with the manuscript terminology.

## Overall

Table S1: Agreement between AI-automated scores and expert scores quantified using MAE, RMSE and ICC for all ethnic groups

| **Facial sign/imaging condition** | **N** | **MAE** | **RMSE** | **ICC (95% CI)** | **%<0.5** | **%<1.0** | **%<1.5** |
| --- | --- | --- | --- | --- | --- | --- | --- |
| Crow's feet | 173 | 0.314 | 0.478 | 0.949 [0.930, 0.960] | 80% | 93% | 98% |
| Eyebags | 173 | 0.184 | 0.365 | 0.906 [0.870, 0.930] | 90% | 96% | 99% |
| Cheek Pig. Spots (Polarized) | 186 | 0.879 | 1.080 | 0.659 [0.130, 0.840] | 31% | 66% | 82% |
| Cheek Pig. Spots (Non-Polarized) | 186 | 0.226 | 0.306 | 0.954 [0.940, 0.970] | 89% | 98% | 100% |
| Cheek Pores | 186 | 0.239 | 0.339 | 0.895 [0.860, 0.920] | 94% | 98% | 99% |
| Corner Lips | 186 | 0.218 | 0.321 | 0.976 [0.970, 0.980] | 91% | 98% | 100% |
| Forehead | 92 | 0.278 | 0.359 | 0.937 [0.910, 0.960] | 87% | 98% | 100% |
| Glabellar | 92 | 0.240 | 0.468 | 0.912 [0.870, 0.940] | 90% | 98% | 99% |
| Inter Ocular | 92 | 0.401 | 0.544 | 0.909 [0.860, 0.940] | 74% | 96% | 99% |
| Nasolabial Fold | 186 | 0.215 | 0.338 | 0.970 [0.960, 0.980] | 88% | 96% | 99% |
| Ptosis | 92 | 0.303 | 0.466 | 0.886 [0.830, 0.920] | 82% | 93% | 99% |
| Underneath Eye | 186 | 0.201 | 0.283 | 0.967 [0.960, 0.970] | 89% | 99% | 100% |
| Upper Lip | 92 | 0.341 | 0.493 | 0.878 [0.820, 0.920] | 76% | 93% | 98% |
| Vascular Disorders (Non-Polarized) | 92 | 0.204 | 0.258 | 0.948 [0.920, 0.970] | 93% | 100% | 100% |
| Vascular Disorders (Polarized) | 92 | 0.177 | 0.218 | 0.963 [0.940, 0.980] | 99% | 100% | 100% |

## East Asian ancestry

Table S2: Agreement between AI-automated scores and expert scores quantified using MAE, RMSE and ICC for east Asian ancestry

| **Facial sign/imaging condition** | **N** | **MAE** | **RMSE** | **ICC (95% CI)** | **%<0.5** | **%<1.0** | **%<1.5** |
| --- | --- | --- | --- | --- | --- | --- | --- |
| Crow's feet | 37 | 0.281 | 0.444 | 0.960 [0.920, 0.980] | 78% | 97% | 97% |
| Eyebags | 37 | 0.205 | 0.372 | 0.932 [0.870, 0.960] | 86% | 97% | 100% |
| Cheek Pig. Spots (Polarized) | 39 | 0.861 | 1.016 | 0.665 [-0.080, 0.890] | 31% | 67% | 87% |
| Cheek Pig. Spots (Non-Polarized) | 39 | 0.251 | 0.350 | 0.930 [0.870, 0.960] | 87% | 95% | 100% |
| Cheek Pores | 39 | 0.157 | 0.185 | 0.967 [0.940, 0.980] | 100% | 100% | 100% |
| Corner Lips | 39 | 0.227 | 0.387 | 0.946 [0.900, 0.970] | 87% | 95% | 100% |
| Forehead | 16 | 0.242 | 0.327 | 0.890 [0.720, 0.960] | 88% | 100% | 100% |
| Glabellar | 16 | 0.177 | 0.235 | 0.961 [0.890, 0.990] | 94% | 100% | 100% |
| Inter Ocular | 16 | 0.380 | 0.439 | 0.949 [0.860, 0.980] | 62% | 100% | 100% |
| Nasolabial Fold | 39 | 0.224 | 0.352 | 0.951 [0.910, 0.970] | 85% | 97% | 100% |
| Ptosis | 16 | 0.269 | 0.367 | 0.935 [0.830, 0.980] | 88% | 94% | 100% |
| Underneath Eye | 39 | 0.208 | 0.288 | 0.965 [0.930, 0.980] | 90% | 100% | 100% |
| Upper Lip | 16 | 0.327 | 0.399 | 0.813 [0.540, 0.930] | 81% | 100% | 100% |
| Vascular Disorders (Non-Polarized) | 16 | 0.145 | 0.209 | 0.761 [0.430, 0.910] | 94% | 100% | 100% |
| Vascular Disorders (Polarized) | 16 | 0.145 | 0.179 | 0.811 [0.540, 0.930] | 100% | 100% | 100% |

## African Ancestry

Table S3: Agreement between AI-automated scores and expert scores quantified using MAE, RMSE and ICC for African ancestry

| **Facial sign/imaging condition** | **N** | **MAE** | **RMSE** | **ICC (95% CI)** | **%<0.5** | **%<1.0** | **%<1.5** |
| --- | --- | --- | --- | --- | --- | --- | --- |
| Crow's feet | 46 | 0.205 | 0.311 | 0.963 [0.930, 0.980] | 93% | 98% | 100% |
| Eyebags | 46 | 0.163 | 0.329 | 0.888 [0.810, 0.940] | 93% | 96% | 100% |
| Cheek Pig. Spots (Polarized) | 50 | 0.990 | 1.220 | 0.599 [0.190, 0.800] | 28% | 66% | 74% |
| Cheek Pig. Spots (Non-Polarized) | 50 | 0.244 | 0.352 | 0.945 [0.900, 0.970] | 84% | 96% | 100% |
| Cheek Pores | 50 | 0.287 | 0.463 | 0.845 [0.730, 0.910] | 90% | 96% | 96% |
| Corner Lips | 50 | 0.178 | 0.268 | 0.975 [0.960, 0.990] | 94% | 98% | 100% |
| Forehead | 26 | 0.298 | 0.410 | 0.954 [0.900, 0.980] | 88% | 92% | 100% |
| Glabellar | 26 | 0.400 | 0.795 | 0.738 [0.500, 0.870] | 77% | 92% | 96% |
| Inter Ocular | 26 | 0.435 | 0.693 | 0.756 [0.530, 0.880] | 77% | 92% | 96% |
| Nasolabial Fold | 50 | 0.221 | 0.340 | 0.973 [0.950, 0.980] | 88% | 94% | 100% |
| Ptosis | 26 | 0.290 | 0.561 | 0.592 [0.270, 0.790] | 81% | 92% | 96% |
| Underneath Eye | 50 | 0.171 | 0.244 | 0.933 [0.890, 0.960] | 94% | 100% | 100% |
| Upper Lip | 26 | 0.236 | 0.378 | 0.684 [0.420, 0.840] | 85% | 96% | 100% |
| Vascular Disorders (Non-Polarized) | 26 | 0.148 | 0.198 | 0.705 [0.430, 0.860] | 100% | 100% | 100% |
| Vascular Disorders (Polarized) | 26 | 0.199 | 0.230 | 0.566 [0.220, 0.780] | 100% | 100% | 100% |

## European ancestry

Table S4: Agreement between AI-automated scores and expert scores quantified using MAE, RMSE and ICC for European ancestry

| **Facial sign/imaging condition** | **N** | **MAE** | **RMSE** | **ICC (95% CI)** | **%<0.5** | **%<1.0** | **%<1.5** |
| --- | --- | --- | --- | --- | --- | --- | --- |
| Crow's feet | 82 | 0.351 | 0.514 | 0.947 [0.920, 0.970] | 78% | 90% | 98% |
| Eyebags | 82 | 0.170 | 0.351 | 0.892 [0.840, 0.930] | 91% | 98% | 98% |
| Cheek Pig. Spots (Polarized) | 91 | 0.828 | 1.022 | 0.697 [0.170, 0.870] | 32% | 66% | 85% |
| Cheek Pig. Spots (Non-Polarized) | 91 | 0.208 | 0.261 | 0.967 [0.950, 0.980] | 92% | 100% | 100% |
| Cheek Pores | 91 | 0.250 | 0.313 | 0.889 [0.840, 0.930] | 92% | 99% | 100% |
| Corner Lips | 91 | 0.238 | 0.323 | 0.979 [0.970, 0.990] | 91% | 99% | 100% |
| Forehead | 47 | 0.278 | 0.341 | 0.930 [0.880, 0.960] | 87% | 100% | 100% |
| Glabellar | 47 | 0.171 | 0.231 | 0.982 [0.970, 0.990] | 96% | 100% | 100% |
| Inter Ocular | 47 | 0.384 | 0.477 | 0.933 [0.880, 0.960] | 77% | 96% | 100% |
| Nasolabial Fold | 91 | 0.214 | 0.340 | 0.974 [0.950, 0.980] | 89% | 97% | 99% |
| Ptosis | 47 | 0.308 | 0.418 | 0.921 [0.860, 0.960] | 81% | 96% | 100% |
| Underneath Eye | 91 | 0.218 | 0.305 | 0.970 [0.960, 0.980] | 86% | 99% | 100% |
| Upper Lip | 47 | 0.392 | 0.567 | 0.880 [0.790, 0.930] | 72% | 89% | 96% |
| Vascular Disorders (Non-Polarized) | 47 | 0.249 | 0.294 | 0.936 [0.880, 0.970] | 91% | 100% | 100% |
| Vascular Disorders (Polarized) | 47 | 0.181 | 0.228 | 0.962 [0.930, 0.980] | 98% | 100% | 100% |
