# Supplementary material for: AI-Based Grading of Facial Aging Signs and Prediction of Visible Age From Standardized Facial Images: Algorithm Development and Internal Evaluation Study
Source: JMIR Dermatol. 2026 Oct 28;9:e93236. doi: 10.2196/93236 (PMC13716349; doi:10.2196/93236)
Supplement: Supplementary file 2 [file derma-v9-e93236-s002.docx]

**Supplementary material II. Bland–Altman Plots**

Bland–Altman plots show the difference between automated and expert scores against their mean. The solid horizontal line represents mean bias; dashed lines represent the 95% limits of agreement.

Figure S1: Bland-Altman plots showing the difference between AI-based (NEXA) and expert scores (y-axis) against their mean (x-axis) for all subjects. The solid black line is the mean bias; dashed red lines are the 95% limits of agreement (bias ± 1.96 SD). Points are color-coded as follows: East Asian ancestry in blue, African ancestry in purple and European ancestry in brown. A horizontal band of points around zero with narrow limits of agreement indicates good interchangeability.
