# Supplementary material for: AI-Based Grading of Facial Aging Signs and Prediction of Visible Age From Standardized Facial Images: Algorithm Development and Internal Evaluation Study
Source: JMIR Dermatol. 2026 Oct 28;9:e93236. doi: 10.2196/93236 (PMC13716349; doi:10.2196/93236)
Supplement: Supplementary file 3 [file derma-v9-e93236-s003.docx]

**Supplementary material III. Calibration Plots**

Calibration plots show automated predictions against expert atlas scores. The identity line represents perfect agreement, and the fitted regression line summarizes calibration.

Figure S2: Calibration plots of AI-predicted scores by NEXA (x-axis) against expert atlas scores (y-axis). Points are color-coded as follows: East Asian ancestry in blue, African ancestry in purple and European ancestry in brown. The dashed diagonal represents perfect agreement (identity line). The solid red line is the fitted ordinary least-squares regression line; the slope and intercept are annotated in each panel. A slope close to 1 and intercept close to 0 indicate well-calibrated predictions.
